# Isolation and characterization of glutathione S-transferase genes and their transcripts in* Saccharina japonica* (Laminariales, Phaeophyceae) during development and under abiotic stress

**DOI:** 10.1186/s12870-023-04430-5

**Published:** 2023-09-18

**Authors:** Chang Lu, Pengyan Zhang, Shuang Li, Mengzhen Cheng, Delin Duan

**Affiliations:** 1 Key Lab of Breeding Biotechnology & Sustainable Aquaculture, Shandong Province Key Laboratory of Experimental Marine Biology, Institute of Oceanology, Chinese Academy of Sciences, Qingdao, 266071 P. R. China; 2 Department of Biological Engineering, College of Life Science, Yantai University, Yantai, 264005 China; 3 Functional Lab for Marine Biology and Biotechnology, National Laboratory for Marine Science and Technology, Qingdao, 266071 China; 4 Division of Mariculture Ecology and Technology, Yellow Sea Fisheries Research Institute, Chinese Academy of Fishery Sciences, Qingdao, 266071 China; 5 University of Chinese Academy of Sciences, Beijing, 100049 P. R. China

**Keywords:** *Saccharina japonica*, Glutathione S-transferase, Transcriptome, Gene expression, Abiotic stresses, Enzyme activity assay

## Abstract

**Background:**

Glutathione S-transferase (GST) is a crucial enzyme for metabolism, detoxification, and stress resistance in organisms. Many GSTs have been identified in seaweeds, but the isolation and functional analysis of GSTs in *Saccharina japonica* have not been completed.

**Result:**

In this study, a total of 32 *SjGST* genes, localized on 10 scaffolds and 6 contigs, were identified and categorized into three groups. Most of these SjGSTs were presumed to be distributed in the cytoplasm. Tandem duplication had a significant influence on the expansion of the *SjGST* gene family. Functional analysis of *cis-*acting elements in the promoter regions demonstrated that *SjGSTs* enhance the stress resistance of the kelp. Quantitative real-time PCR tests confirmed that *SjGSTs* positively influence *S. japonica* sporophytes under stress from low salinity, drought, and high temperature. Recombinant yeast tests further affirmed the role of SjGSTs in stress resistance; SjGSTs improved the growth rate of recombinant yeast under 1.5 M NaCl or 8 mM H_2_O_2_. Analysis of biochemical parameters indicated that the optimum temperatures for SjGST20 and SjGST22 were 20 °C, and the optimum pH values were 7.0 and 8.0 for SjGST20 and SjGST22, respectively. The *K*_*m*_ values for the substrate 1-chloro-2,4-dinitrobenzene (CDNB) were 2.706 mM and 0.674 mM and were 6.146 mM and 3.559 mM for the substrate glutathione (GSH) for SjGST20 and SjGST22, respectively.

**Conclusion:**

*SjGSTs* are important stress resistant genes in *S. japonica*. This research results will enhance our understanding the function of GSTs in brown seaweeds, and explained its functional roles in stress resistance in marine environments.

**Supplementary Information:**

The online version contains supplementary material available at 10.1186/s12870-023-04430-5.

## Background

Glutathione S-transferase (GST, EC 2.5.1.18) is a functional enzyme found in a wide variety of species including plants, animals, bacteria, and algae [1, 2]. Based on their subcellular localization inside a mammalian cell and their structures, GSTs can be divided into three categories: cytosolic GSTs, mitochondrial GSTs (kappa-class GSTs), and microsomal GSTs (Membrane-Associated Proteins in Eicosanoid and Glutathione metabolism, MAPEG) [3, 4].

Cytosolic GSTs are the most abundant GSTs in cells and exist as dimers [4]. The molecular weight of each subunit ranges from 23 to 29 kDa [5]. Based on their origin, sequence similarity, amino acids in active sites, and substrate specificity, cytosolic GSTs can be divided into the following categories: alpha, beta, delta, epsilon, zeta, theta, mu, nu, pi, sigma, tau, phi, and omega [4, 6]. Structurally, the cytosolic GSTs and mitochondrial GSTs have high similarities, while microsomal GSTs are quite different [7]. A typical GST subunit contains two active domains. The thioredoxin-folded N-terminal domain is highly conserved and specifically binds to the substrate glutathione (GSH), while the C-terminal domain determines substrate specificity [8].

GSTs are originally discovered as a detoxifying enzyme to reduce the adverse effects of toxic substances on organisms [9]. They detoxicate by catalyzing the conjugation of toxic endogenous and exogenous electrophilic compounds with reduced GSH, which serves as a nucleophile [10]. This GST-catalyzed enzymatic reaction effectively increases the solubility of non-polar toxic substances and promotes further metabolic processing [11]. GSTs also play important roles in plant resistance to abiotic stresses, effectively scavenging excessive reactive oxygen species (ROS), and alleviating oxidative stress in plants [12]. For example, overexpression of *OsGSTU5* gene improved the tolerance of rice to toxic heavy metal ion arsenic and effectively limited the upward transport of arsenic within the plant [13].The GST genes of *Hordeum vulgare*, *Populus trichocarpa*, *Vitis vinifera*, *Camellia sinensis*, and other species were overexpressed in *Arabidopsis*, and the growth status and physiological indicators of transgenic plants under salt, drought, and temperature stresses were better than those of wild type *Arabidopsis* [14–19]. A jasmonate-responsive *GST* can improve the ability of tomatoes to resist cold stress [20]. Overall, genome-wide identification and functional verification have been conducted on GST genes in many plants, including *Solanum tuberosum*, *Gossypium raimondii* and *Cucurbita maxima* [21–23]. Research results indicated that GSTs are indispensable functional enzymes involved in a variety of processes including plant growth and development, hormone signal transduction, cell signal transduction and regulation, biosynthesis of secondary metabolites, and response to biotic and abiotic stresses [24].

*Saccharina japonica* is one of the most economically important seaweeds in China. In addition to being rich in nutrients, the polysaccharides contained in the sporophytes, such as alginate and fucoidan, have great potential for development and utilization in many fields including industry, medicine, and food [25, 26]. However, the growth of *S. japonica* is predictably affected by environmental factors, such as temperature, diseases, and heavy metal ions, resulting in the reduction of yield. Therefore, improving the resistance of *S. japonica* to stress will have significant positive effects on its growth and yield.

To date, many studies have shown that GST in algae can actively respond to abiotic stresses. The stimulation of algae by plant hormones, pH, temperature, desiccation, heavy metal ions, salinity and organic pollutants can increase GST transcription level or activity [27–31]. In the red algae *Chondrus crispus*, herbicides and insecticides strongly induced the expression of GSTs [32]. In the model brown algae *Ectocarpus siliculosus*, both the expression level and the total activity of GST were higher than in the control group after treatment with copper ion and glyphosate, respectively [2]. However, there is a lack of in-depth research on the *GST* gene in algae, especially for *S. japonica*, the structure and behavior of GSTs have not been well studied.

In this study, 32 *SjGST* genes were identified from the whole *S. japonica* genome. Features of their sequences and functions were characterized. The transcriptional profiles under low salinity, drought, and high temperature and throughout the developmental stages of *S. japonica* were also analyzed. This study reveals the role of *SjGSTs* when *S. japonica* sporophytes suffer from abiotic stresses.

## Results

### Identification and characterization of *SjGST* sequences

In total, there were 32 *GST* sequences deduced from the *S. japonica* genome (MEHQ0000000), which were named successively *SjGST1* to *SjGST32*. The name, gene ID, scaffold location, open reading frame (ORF) length, exon number, amino acid number, molecular weight (MW) and isoelectric point (pI) of the *SjGSTs* are summarized in Table 1.

**Table 1:** The deduced *SjGSTs* and their biochemical features

ID	Gene	pI	MW (KDa)	ORF	Amino acid	Exon	Scaffolds	Genomic location	Sub-family class
GENE_000958	SjGST1	4.76	10.73	303	100	3	Contig4725	16,858–18,884	Sigma
GENE_003591	SjGST2	6.53	12.04	333	110	5	chr10	2,365,817–2,370,700	Sigma
GENE_003790	SjGST3	6.36	42.47	1146	381	7	chr16	6,463,588–6,467,317	Omega
GENE_004442	SjGST4	4.61	30.74	858	285	6	chr26	6,915,485–6,934,639	-
GENE_006883	SjGST5	8.48	22.88	630	209	2	Contig1585	849,708–851,175	-
GENE_007480	SjGST6	4.99	22.92	618	205	6	Contig1161	96,559–100,943	Sigma
GENE_010113	SjGST7	4.58	22.58	618	205	6	chr2	988,823–1,005,443	Sigma
GENE_011072	SjGST8	8.81	35.87	984	327	8	chr8	13,793,309–13,799,350	-
GENE_011958	SjGST9	5.21	47.72	1293	430	12	chr12	586,758–593,609	-
GENE_013030	SjGST10	6.81	31.03	834	277	5	Contig3114	37,142–45,054	Sigma
GENE_013031	SjGST11	4.3	12.22	336	111	2	Contig3114	24,790–27,835	Sigma
GENE_013032	SjGST12	4.3	22.04	606	201	5	Contig3114	9108–12,545	Sigma
GENE_015111	SjGST13	4.79	21.78	606	201	5	chr2	13,725,140–13,731,638	Sigma
GENE_015113	SjGST14	4.6	21.73	606	201	5	chr2	13,899,272–13,904,873	Sigma
GENE_015114	SjGST15	4.46	21.82	606	201	5	chr2	13,883,761–13,891,137	Sigma
GENE_015124	SjGST16	4.48	22.33	606	201	5	chr2	13,907,979–13,920,587	Sigma
GENE_015125	SjGST17	4.8	16.32	456	151	4	chr2	13,781,933–13,784,655	-
GENE_015126	SjGST18	4.54	21.84	606	201	5	chr2	13,738,092–13,743,179	Sigma
GENE_015127	SjGST19	4.65	21.82	606	201	5	chr2	13,937,559–13,943,219	-
GENE_018185	SjGST20	4.87	21.71	606	201	5	chr18	12,985,160–12,990,817	-
GENE_018444	SjGST21	4.93	23.8	666	221	6	Contig2153	52,647–57,995	Sigma
GENE_020841	SjGST22	4.92	22.31	618	205	6	chr11	10,064,826–10,072,916	Sigma
GENE_020842	SjGST23	6.93	14.3	387	128	4	chr11	9,970,276–9,975,317	-
GENE_020843	SjGST24	4.52	10.82	300	99	3	chr11	10,026,244–10,030,775	-
GENE_021351	SjGST25	7.34	26.87	726	241	6	chr14	6,981,619–7,001,362	Sigma
GENE_022334	SjGST26	5.75	23.78	663	220	6	chr16	4,389,593–4,394,899	Sigma
GENE_023686	SjGST27	4.51	14.17	372	123	3	chr22	4,499,841–4,502,646	-
GENE_023690	SjGST28	8.58	23.21	627	208	5	chr22	4,504,960–4,511,106	-
XLOC_014659	SjGST29	9.08	9.18	258	85	2	Contig2153	128,237–137,460	Sigma
XLOC_028480	SjGST30	6.25	14.45	408	135	3	Contig4725	986–3572	Sigma
XLOC_034827	SjGST31	4.7	10.95	309	102	2	chr2	10,490,301–10,494,815	-
XLOC_036550	SjGST32	9.19	23.84	660	219	6	Contig847	47,142–58,410	-

The numbers of amino acids of the SjGSTs ranged from 85 to 430, and their MW from 9.18 kDa to 47.72 kDa for SjGST29 and SjGST9, respectively. The predicted pI values of the SjGST proteins ranged from 4.3 (SjGST11 and SjGST12) to 11.41 (SjGST30). Based on SjGSTs sequence alignment results in NCBI Conserved Domain, 18 of the 32 SjGSTs were sigma_class, SjGST3 was omega_class, others have not been classified yet (Table 1). The predicted localization of the SjGST proteins showed that most of the SjGSTs were localized in the cytoplasm (Additional file 1: Table S1). However, SjGST8 was localized in the nucleus, SjGST27 in the chloroplast, and SjGST31 extracellularly. Further, SjGST4 had a transmembrane helix. Three SjGSTs (SjGST4, SjGST5, and SjGST8) had signal peptides.

### Phylogenetic, motif, and gene structure analysis of the *SjGST* genes

**Fig. 1:**
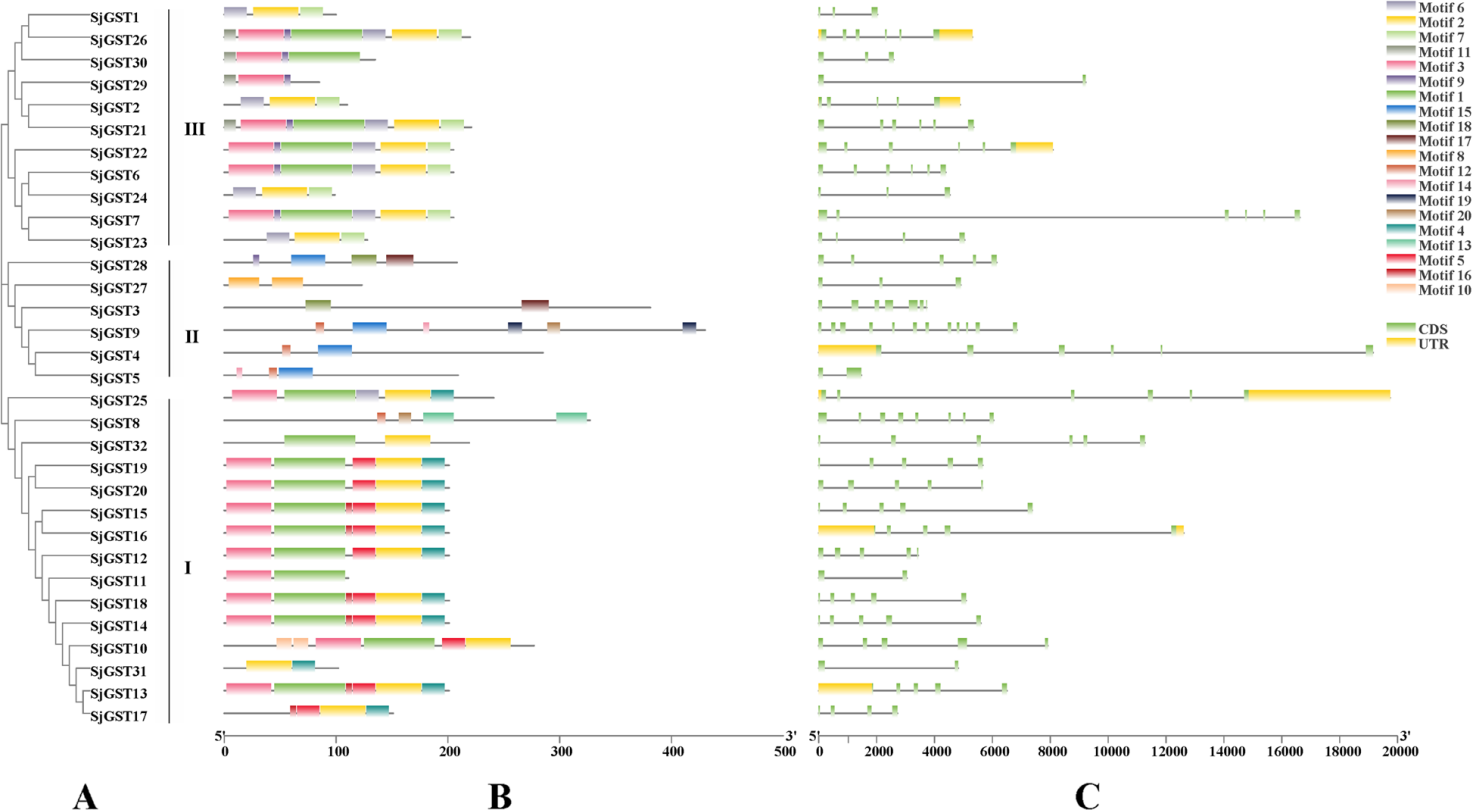
Phylogenetic relationships, conserved motifs, and gene structure of the SjGSTs in *S.japonica*. **A** The maximum likelihood phylogenetic tree of the 32 SjGST proteins. **B** Conserved motifs identified in the 32 SjGST proteins. **C** Structure of the *SjGST* genes. Exons, introns, and untranslated regions (UTRs) are indicated by blue boxes, blank lines, and grey boxes, respectively

According to the conserved motifs and location in the Maximum likelihood (ML) phylogenetic tree, the 32 *S. japonica* GST proteins primarily could be classified into three groups (I-III) (Fig. 1A). The result of reciprocal BLAST suggested that sequences in group I and III exhibited homology (Additional file 2: Table S2). A total of 20 conserved motifs, whose lengths ranged from 6 to 65 amino acids, were identified by MEME (Fig. 1B and Additional file 3: Table S3). *SjGST* sequences in the same group had similar types and compositions of motifs. Motifs 1, 2, and 3 were found in groups I and III. Motif 5 was unique to group I, motif 8 to group II, and motif 7 to group III. Motifs 2, 3, 4, 8, 13 and 15 had GST conserved regions. The number of introns in the *SjGSTs* ranged from 1 to 11. Each sequence contained approximately 4 introns on average. The longest intron identified among the *SjGSTs* was nearly 13 kb (Fig. 1C and Table 1).

**Fig. 2:**
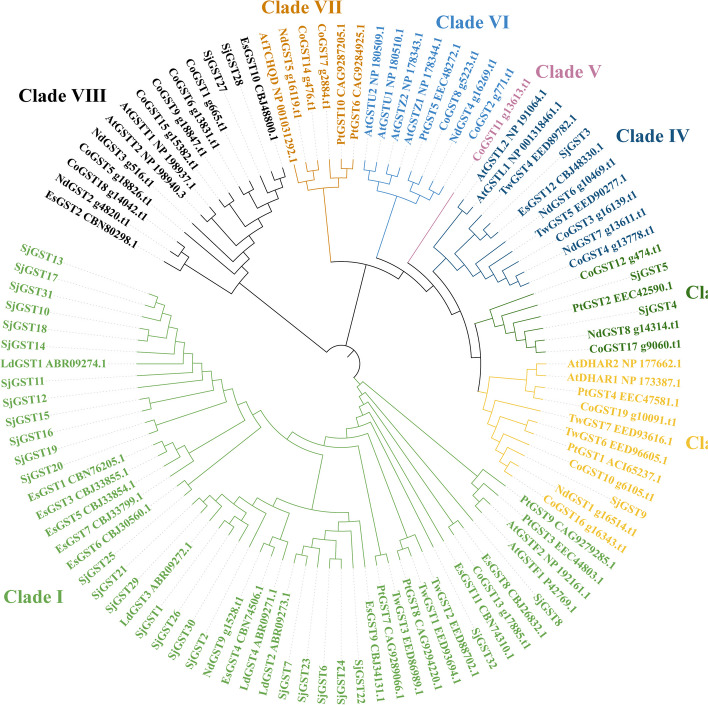
The maximum likelihood phylogenetic tree of the GST proteins from *Ectocarpus siliculosus*, *Cladosiphon okamuranus*, *Nemacystus decipiens*, *Laminaria digitata*, *Phaeodactylum tricornutum, Thalassiosira weissflogii, Arabidopsis thaliana* and *S. japonica*

A ML phylogenetic tree was constructed set *Arabidopsis* as outgroup. The 106 GST sequences from the brown algae (*S. japonica*, *Ectocarpus siliculosus*, *Cladosiphon okamuranus*, *Nemacystus decipiens*, *Laminaria digitata*), diatoms (*Phaeodactylum tricornutum*, *Thalassiosira weissflogii*), and green plant (*Arabidopsis thaliana*) were divided into 8 clades (I-VIII) (Fig. 2 and Additional file 4: Table S4). Groups I and III of the 32 SjGSTs in Fig. 1 were clustered into clades I. These 24 SjGSTs showed a closer evolutionary relationship with *E. siliculosus* and *L. digitata* than with other species. The sequences in group II were scattered among other clades, may be homologous to CoGSTs (Additional file 2: Table S2). Based on the conserved domains, most of the algae sequences in clade I were sigma class GSTs, in clade IV were omega class GSTs. CoGST1, CoGST6, CoGST9, and EsGST10 in clade VIII were Ure2p subfamily (Additional file 4: Table S4).

### Alternative splicing analysis of the *SjGSTs*

The types and numbers of alternative splicing (AS) sites in the *SjGSTs* in different tissues and developmental stages were analyzed. Transcription start sites (TSS) were the most abundant AS site type, followed by transcription terminal sites (TTS); other types included were exon skipping (ES), p3_splices, p5_splices, and intron retention (IR). Details of these sites are listed in Additional file 5: Table S5.

### Scaffold locations and gene duplications of the *SjGSTs*

**Fig. 3:**
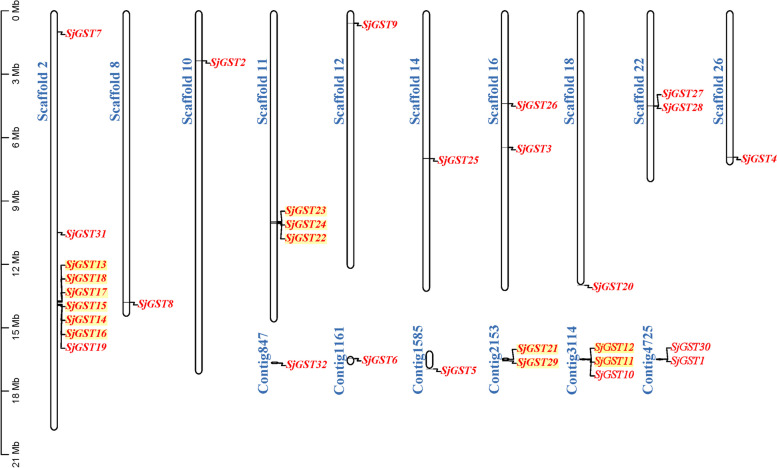
Scaffold locations of the *SjGST* genes and their duplications. Tandem duplicated genes are indicated by a yellow background

The 32 *SjGSTs* were localized on 10 scaffolds and 6 contigs. Four tandem duplications were found (*SjGST13-18*; *SjGST21* and *SjGST29*; *SjGST11* and *SjGST12*; and *SjGST22-24*). Except for the genes that were tandemly duplicated, other genes rarely clustered (Fig. 3).

### Secondary structure features of the SjGSTs

**Fig. 4:**
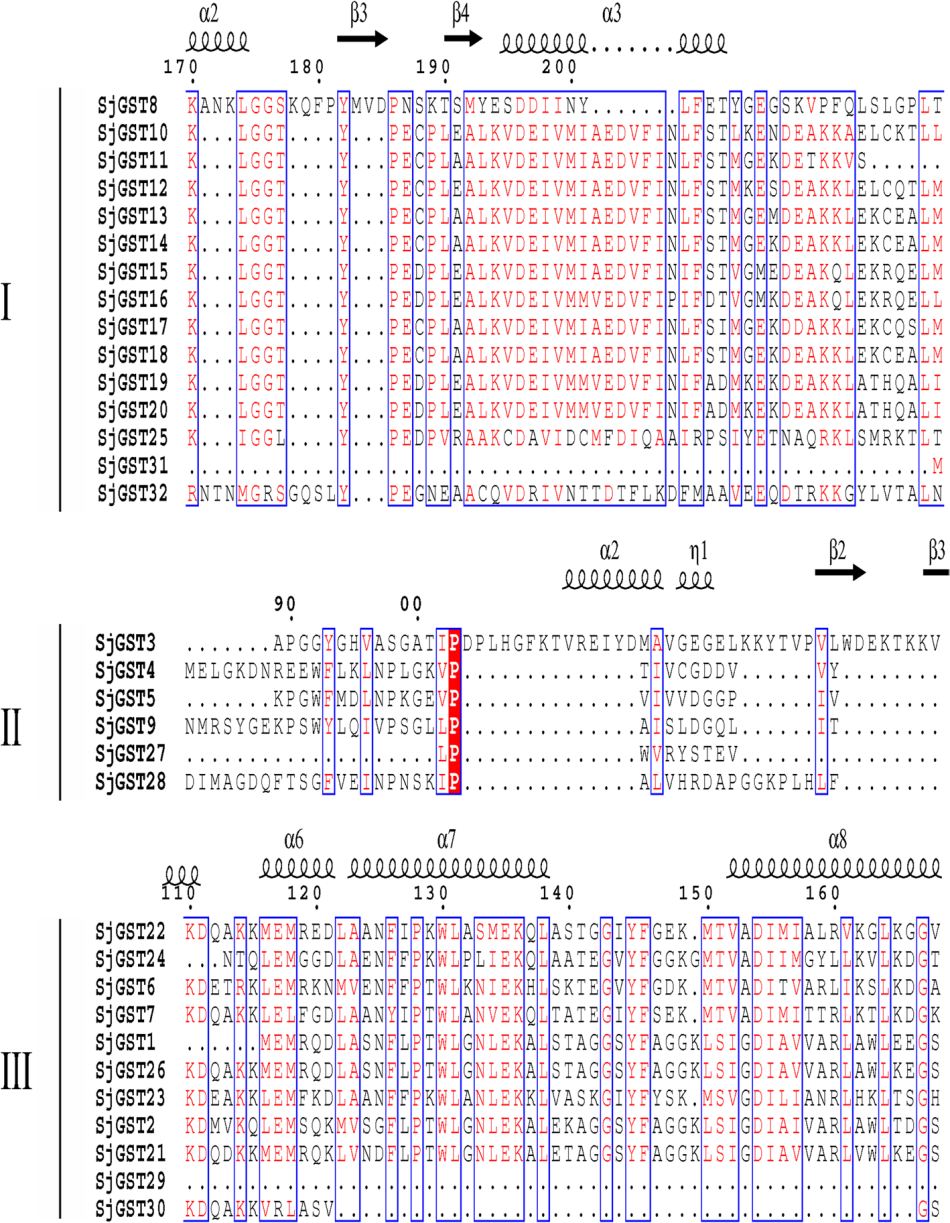
Representative secondary structures and sequence alignment of the SjGSTs. The secondary structures of the SjGSTs are shown above the alignment. α-helices are represented by helix symbols, β-sheets with arrows, and β-turns with the letters TT

According to the self-optimized prediction method with alignment (SOPMA) results, there were four kinds of secondary structures in SjGST amino acid sequences: α-helices were the primary secondary structures, followed by random coils, β-sheets, and β-turns (Additional file 6: Table S6). Figure 4 illustrates the representative conserved amino acid sites and secondary structures.

### Transcriptional profiles of *SjGSTs* in different tissues and developmental stages

**Fig. 5:**
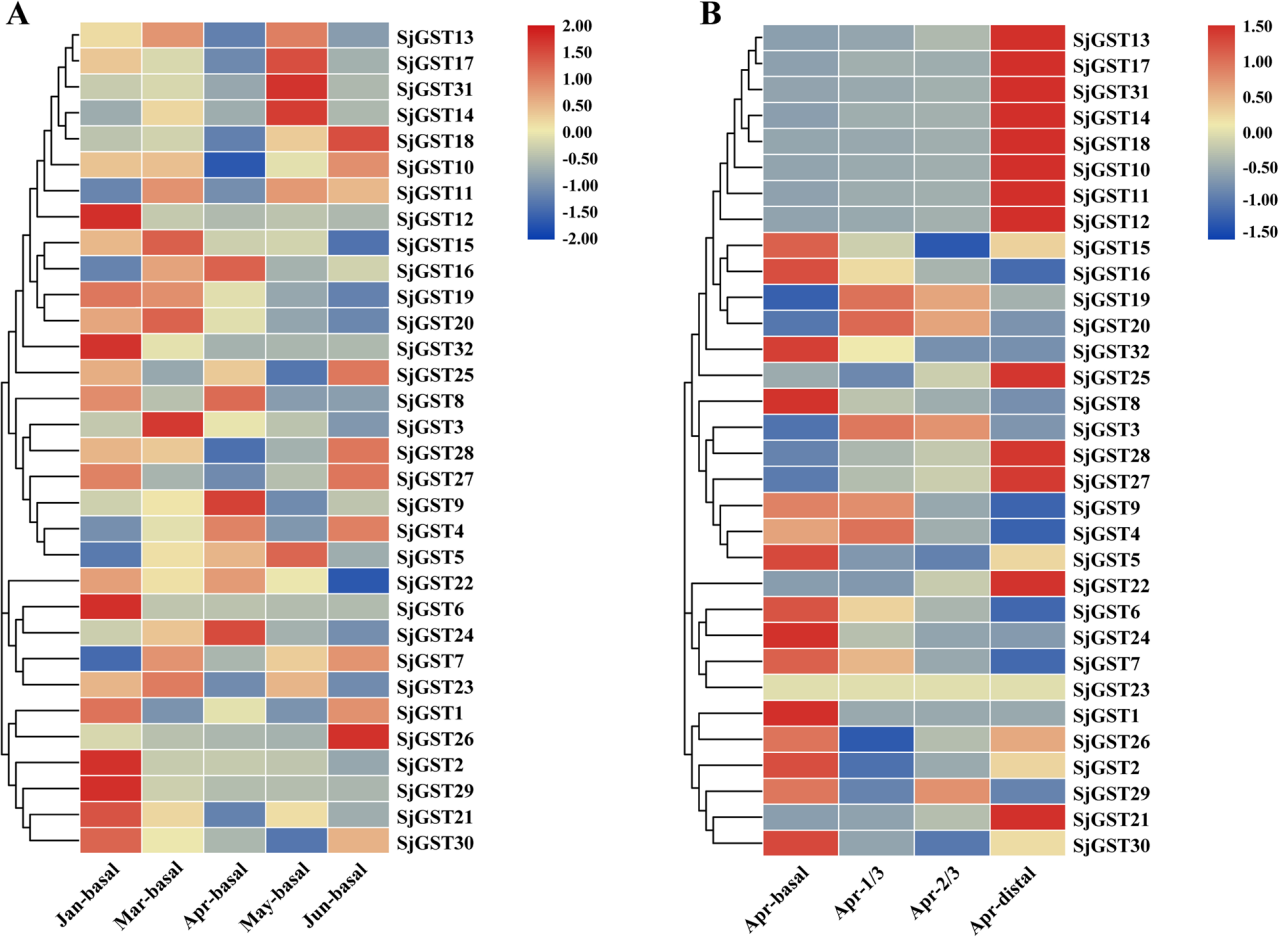
Transcriptions profiles of *S. japonica SjGSTs*. **A** *SjGSTs* transcripts in different developmental stages. **B** *SjGSTs* transcripts in different tissues. Jan: January, Mar: March, Apr: April, Jun: June

TBtools was used to draw a heatmap showing changes in the transcription levels of *SjGSTs* under various developmental stages (Fig. 5A) and tissues (Fig. 5B) [33]. During the growth and development of *S. japonica*, a total of 25 *SjGSTs* were differentially expressed. The expression levels of most group III *SjGSTs* (Fig. 1A) decreased, while other *SjGSTs* had the highest expression level in March to May. Several *SjGSTs*, such as *SjGST7*, *SjGST20*, *SjGST22* had high expression levels throughout development, but *SjGST1* and *SjGST32* were almost not expressed.

In different tested tissues, a total of 21 differentially expressed *SjGSTs* were detected. Within the same subgroup (Fig. 1A), gene expression trends were clearly similar. For example, from basal blade to distal blade, the expression levels of most sequences in group I increased, while group III expression levels decreased. Some *SjGSTs* had consistently high expression levels in different tissues, such as *SjGST16* and *SjGST22*, while some were only expressed in specific tissues like *SjGST11* (highly expressed in basal blades) and *SjGST31* (highly expressed in basal blades).

In order to more intuitively display the expression level change trends of *SjGST* genes, STEM software was used for expression pattern analysis [34]. Tables 2 and 3 showed the profiles of *SjGSTs* expression patterns in different development stages and sporophyte tissues, respectively. Some genes are not listed in Table 2 (like *SjGST4*) or Table 3 (like *SjGST1*) because their expression changes in the tested samples are not different.

**Table 2:**
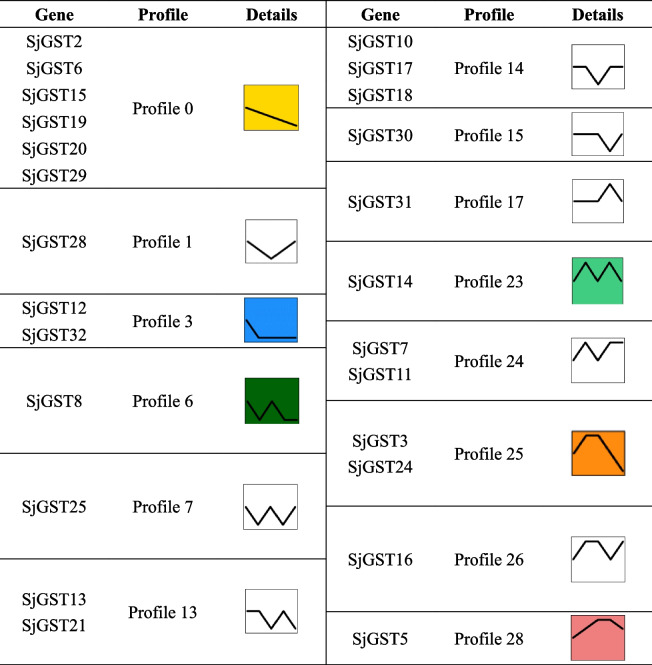
Transcriptional profiles of *SjGSTs* in *S. japonica* at different development stages

**Table 3:**
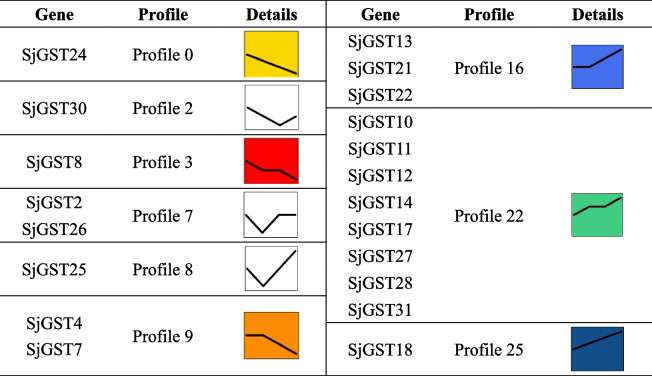
Transcriptional profiles of *SjGSTs* in *S. japonica* in different tissues

From January to June, the differentially expressed *SjGSTs* exhibited six significant expression patterns: profile 0 (*SjGST2*, *SjGST6*, *SjGST15*, *SjGST19*, *SjGST20*, and *SjGST29*), profile 3 (*SjGST12* and *SjGST32*), profile 8 (*SjGST8*), profile 23 (*SjGST14*), profile 25 (*SjGST3* and *SjGST24*), and profile 28 (*SjGST5*). Profiles 0, 3, 6, and 25 exhibited down-regulated expression trends; profile 0 was a representative profile. Genes involved in oxidative phosphorylation, photosynthesis, carbon fixation, and metabolic pathways were enriched in these profiles (Table 2 and Additional file 7: Table S7).

For the different tissues (basal blade, 1/3 blade, 2/3 blade and distal blade), of *S. japonica* sporophytes, the significant expression patterns of the differentially expressed *SjGSTs* were profile 0 (*SjGST24*), profile 3 (*SjGST8*), profile 9 (*SjGST4*, *SjGST7*, and *SjGST9*), profile 16 (*SjGST13*, *SjGST21*, and *SjGST22*), profile 22 (*SjGST10*, *SjGST11*, *SjGST12*, *SjGST14*, *SjGST17*, *SjGST27*, *SjGST28*, and *SjGST31*), and profile 25 (*SjGST18*). Profiles 0, 3, and 9 exhibited down-regulated expression trends from the basal blade to the distal blade, while profiles 16, 22, and 25 were up-regulated. These profiles were enriched in genes related to biological metabolism, biosynthesis, and photosynthesis (Table 3 and Additional file 8: Table S8.).

According to the Kyoto Encyclopedia of Genes and Genomes (KEGG) pathway enrichment analysis, most *SjGSTs* functioned in a metabolism pathway, including glutathione metabolism and arachidonic acid metabolism (except for *SjGST3*, *SjGST8*, *SjGST11*, *SjGST27*, *SjGST28*, *SjGST29*, *SjGST31*, and *SjGST32*) (Additional file 9: Table S9.).

### Stress-related *cis*-acting elements in *SjGST* promoters

**Fig. 6:**
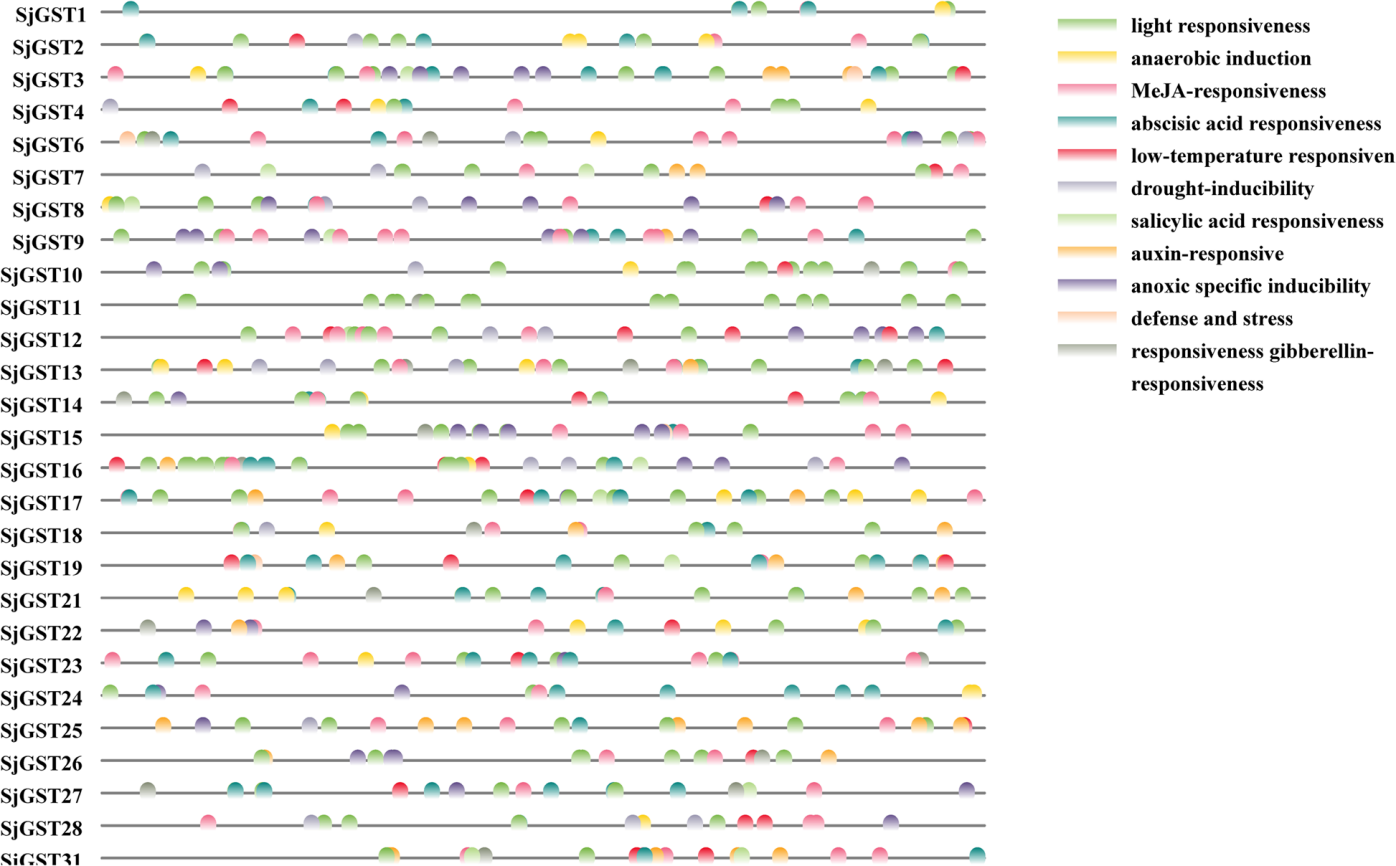
The *cis-*acting elements responding to abiotic stress in the 2-kb promoter regions of *SjGSTs*

*Cis-*acting elements localized in upstream sequences of the translation start sites of *SjGSTs* could illustrate the potential regulatory mechanisms of *SjGSTs* during abiotic stress responses. To detect *cis-*acting elements, 2-kb up-stream sequences of 28 *SjGSTs* (promoter regions for *SjGST5*, *SjGST20*, *SjGST29*, and *SjGST30* were absent) were extracted and analyzed with PlantCARE. A total of 11 kinds of *cis-*acting elements were detected, which were involved with responses to light, methyl jasmonate (MeJA), abscisic acid, low temperature, salicylic acid, auxin, defense, stress, and gibberellin, anaerobic induction, drought inducibility, and anoxic-specific inducibility (Fig. 6).

### Transcriptional profiles of *SjGST* genes under abiotic stresses

**Fig. 7:**
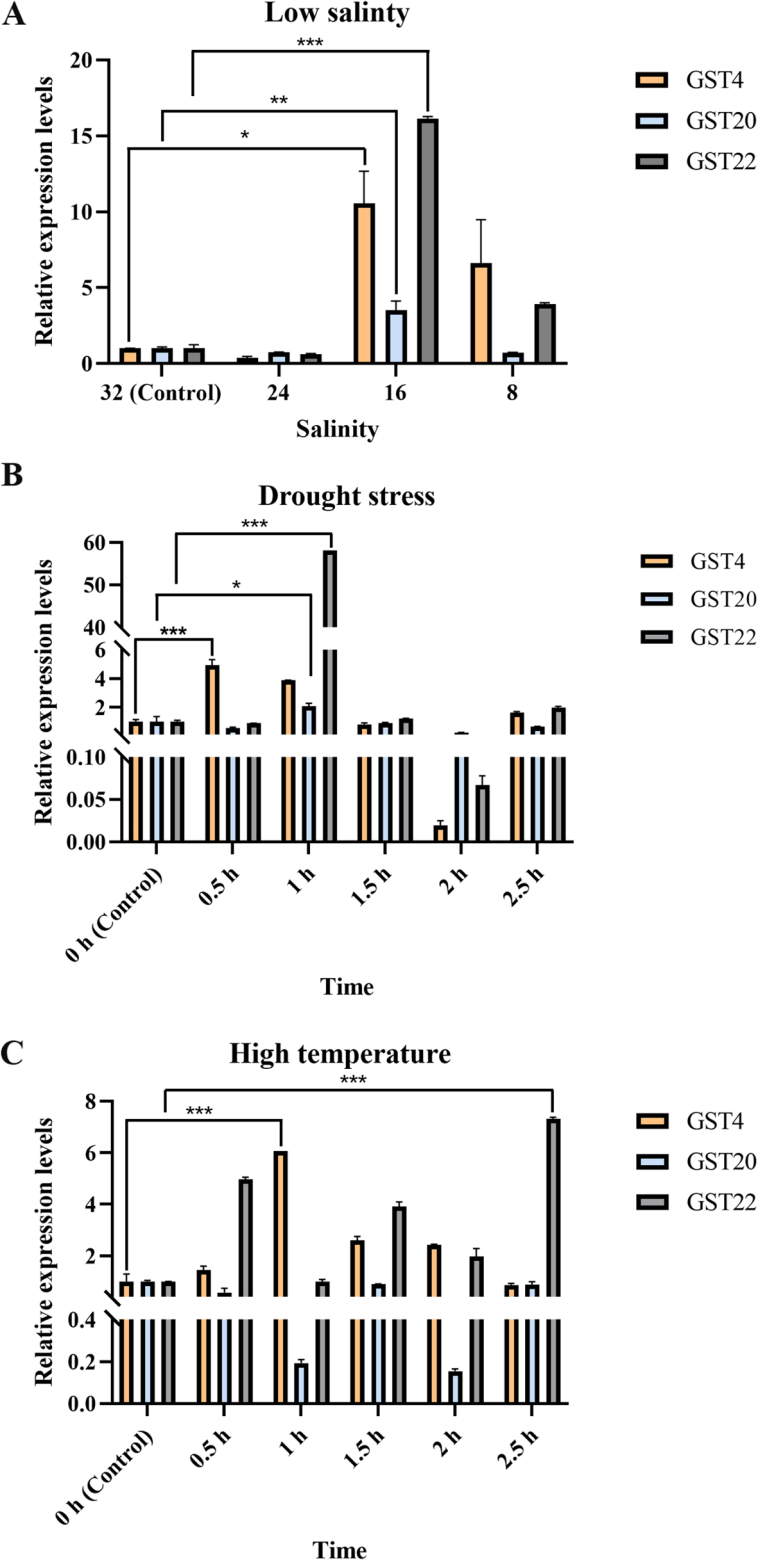
Relative expression levels of *SjGST4*, *SjGST20*, and *SjGST22* under abiotic stresses. **A** Expression profiles of *SjGSTs* under 24‰, 16‰, 8‰ low salinity stress. **B** Expression profiles of *SjGSTs* under drought. **C** Expression profiles of *SjGSTs* under 25 °C high temperature stress. *P < 0.05, **P < 0.01, and ***P < 0.001

As shown in Fig. 7, *SjGST4*, *SjGST20*, and *SjGST22* were observed to be up-regulated under low salinity, drought, and high temperature, indicating that they were involved in the response to stresses. For the low-salinity treatments, the gene expression level under 32‰ salinity was set as the control. After treated for 1.5 h, *SjGST4*, *SjGST20*, and *SjGST22* reached their highest expression levels of expression under salinity 16‰, which were respectively 10.5-fold, 3.5-fold, and 16.1-fold greater than control (Fig. 7A).

For drought stress, the expression levels of both *SjGST4*, *SjGST20* and *SjGST22* increased significantly (*p* < 0.05) (Fig. 7B). Among them, *SjGST4* was the first to reach the peak, which expression level increased 4.9-fold than control. Under treated 1 h, both the expression level of *SjGST20* (2.1-fold) and *SjGST22* (58-fold) were the highest. Overall, the expression trend of these three *SjGSTs* under drought treatment was the same. After peak, their expression levels decreased with the increase of treatment time, but slightly increased at 2.5 h. After high temperature stress (25℃) for 1 h, *SjGST4* reached its highest expression level, which was 6.1-fold higher than the control. The expression of SjGST22 fluctuated at different time points, and eventually reached a peak at 2.5 h. Its expression level increased 7.3-fold. The expression level of *SjGST20* was lower than the control at each time point (Fig. 7C).

According to the experimental results, these three sequences had varying degrees of response to low salinity, drought, and high temperature. Drought caused the strongest changes in gene expression. Compared to *SjGST4* and *SjGST20*, *SjGST22* was more sensitive to environmental changes.

### Growth of recombinant yeast under abiotic stresses

**Fig. 8:**
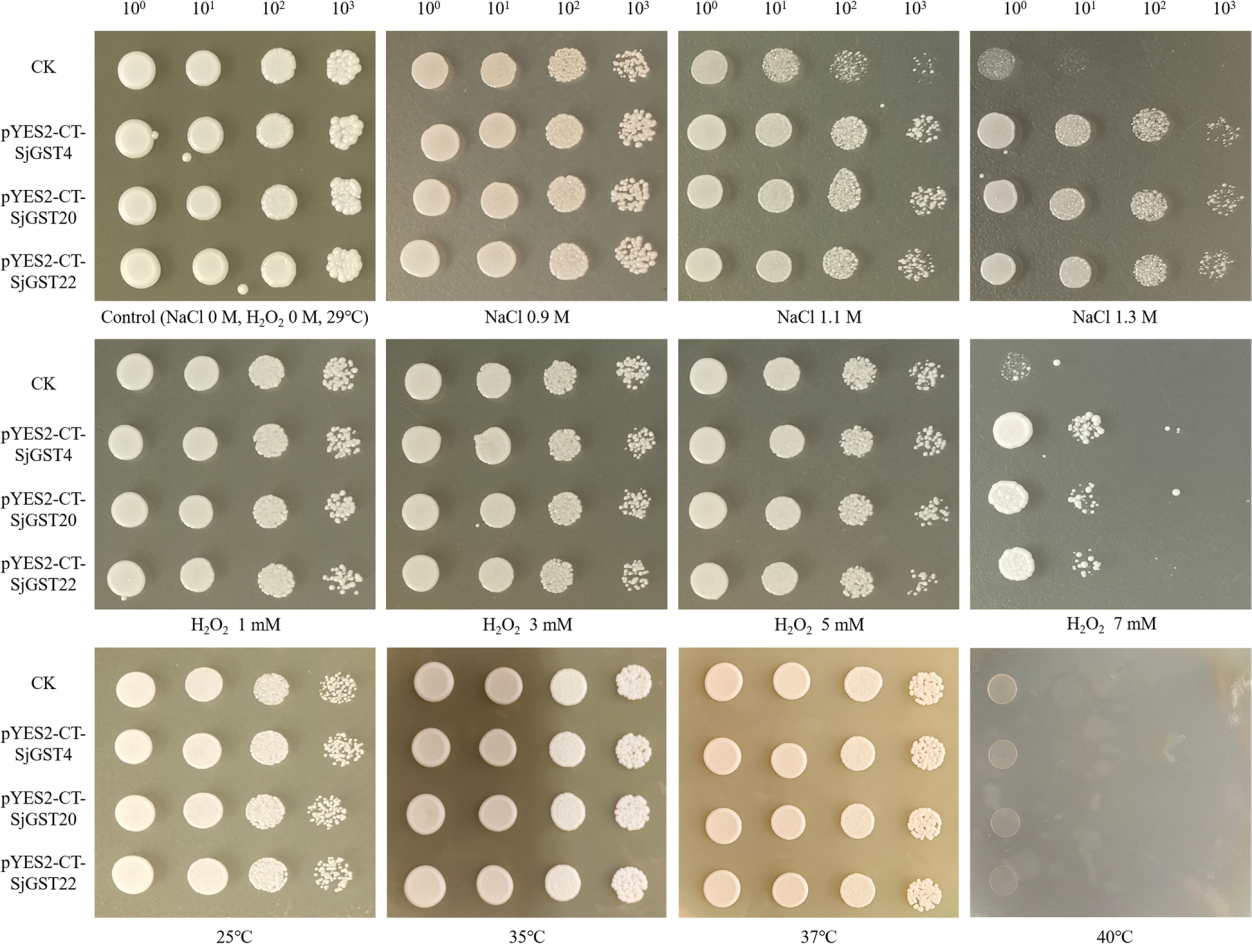
The growth pattern of CK and recombinant strain pYES2-CT-SjGST4, pYES2-CT-SjGST20 and pYES2-CT-SjGST22 on YPD plates for 4 d at different concentration NaCl, H_2_O_2_ and temperatures. CK: INVSc1 transformed with an empty pYES2-CT vector. Control: YPD plate with 0 M NaCl and 0 M H_2_O_2_ cultured under 29℃. 10^0^–10^3^: dilution ratio of yeast culture solution

To further illustrate the *SjGSTs* response to stress resistance, observed the growth of recombinant yeast under NaCl, H_2_O_2_, and temperature stresses. On YPD control plates (0 M NaCl, 0 M H_2_O_2_, 29℃), there was no difference in growth rate between CK (INVSc1 transformed with an empty pYES2-CT vector) and recombinant yeast pYES2-CT-SjGST4, pYES2-CT-SjGST20 and pYES2-CT-SjGST22 (Fig. 8), which demonstrated that inserted *SjGSTs* did not affect the normal growth of *Saccharomyces cerevisiae*.

Cultured under 29℃, with increased NaCl or H_2_O_2_ concentrations, the growth of recombinant yeast was faster than CK. This difference in growth was particularly significant under 1.5 M sodium chloride and 8 mM H_2_O_2_ treatments. On these two plates, CK colonies cannot be observed, while the colonies of recombinant yeast were obvious. For the temperature stresses, compared with the optimum growth temperature 29℃, at 25℃, 35℃ and 37℃, the growth of CK and recombinant yeast was approximately the same, while at 40℃, yeast in all the groups unable grow normally, which were inhibited by high temperature. The above results indicated that SjGSTs participated in the stress of s NaCl and H_2_O_2_, improving the viability of recombinant yeast under adverse conditions.

### Analysis of SjGST20 and SjGST22 enzymatic activities

**Fig. 9:**
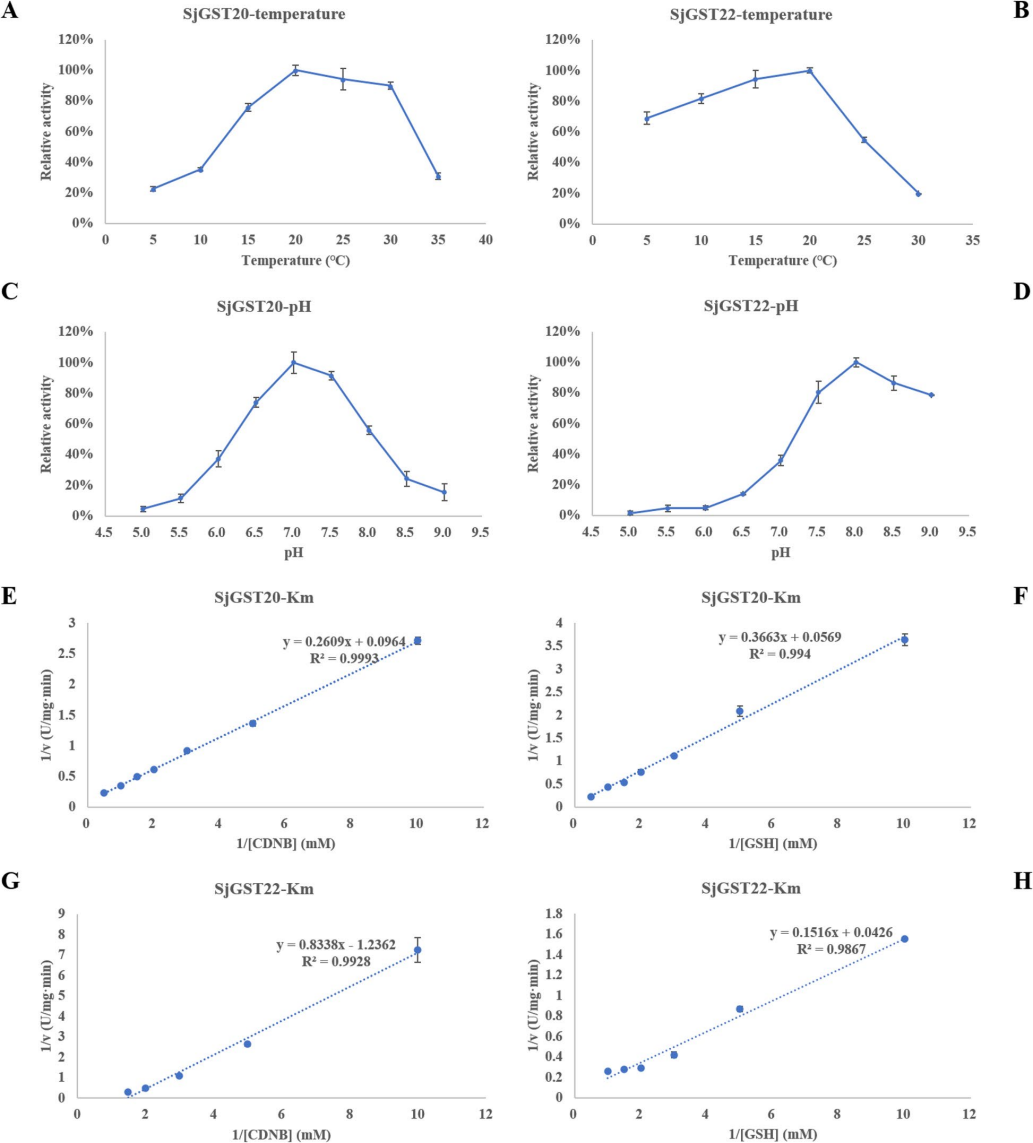
Optional temperature, pH, and *K*_*m*_ for CDNB and GSH of SjGST20 and SjGST22. **A** Influence of temperature (5–35 °C) on the activity of SjGST20. **B** Influence of temperature (5–30 °C) on the activity of SjGST22. **C** Influence of pH (5.0–9.0) on the activity of SjGST20. **D** Influence of pH (5.0–9.0) on the activity of SjGST22. **E** The Lineweaver–Burk plots of SjGST20 for the substrate CDNB. **F** The Lineweaver–Burk plots of SjGST20 for the substrate GSH. **G** The Lineweaver–Burk plots of SjGST22 for the substrate CDNB. **H** The Lineweaver–Burk plots of SjGST22 for the substrate GSH

SjGST20 and SjGST22 showed the highest enzyme activity in the enzyme activity detection experiment of crude recombinant protein, so they were selected to explore enzymatic properties. After purification through the His-Ni^2+^ column, the MW of SjGST20 and SjGST22 fusion proteins were about 41.8 kDa and 42.4 kDa, respectively, which were consistent with the prediction (Additional file 10: Figure S1 and Additional file 11: Figure S2). The enzyme activities of the recombinant proteins were determined. The optimal temperature for the activity of SjGST20 and SjGST22 was 20 °C (Fig. 9A and B). Both recombinant enzymes were sensitive to high temperatures. The optimal pH values were 7.0 and 8.0 for the activity of SjGST20 and SjGST22, respectively (Fig. 9C and D). SjGST20 activity was affected by acidic and basic pH, while SjGST22 was almost inactivated under acidic pH but had high tolerance to basic pH.

It is specified that under the optimum temperature and pH conditions, in one minute per milligram protein is catalyzed 1 μmol CDNB combines with GSH as an enzyme activity unit. Using a typical Michaelis–Menten kinetics analysis, the kinetic parameters of the recombinant enzymes were determined. The *K*_*m*_ values of SjGST20 were 2.706 mM for CDNB and 6.146 mM for GSH (Fig. 9E and F), while the *K*_*m*_ values of SjGST22 were 0.674 mM for CDNB and 3.559 mM for GSH (Fig. 9G and H). Enzyme kinetic parameters of SjGST20 and SjGST22 are listed in Table 4, including *V*_*max*_, *K*_*cat*_ and *K*_*cat*_ /* K*_*m*_.

**Table 4:** Enzyme kinetic parameters of SjGST20 and SjGST22

**Substrate**	**SjGST20**		**SjGST22**	
	**CDNB**	**GSH**	**CDNB**	**GSH**
*K*_*m*_ (mM)	2.706	6.146	0.674	3.559
*V*_*max*_ (U mg^−1^ min^−1^)	10.373	16.779	0.809	23.474
*K*_*cat*_ (s^−1^)	9.829	15.898	0.478	13.883
*K*_*cat*_ / *K*_*m*_ (mM^−1^ s^−1^)	3.632	2.586	0.709	3.901

Both SjGST20 and SjGST22 had a higher affinity for CDNB than GSH. *K*_*m*_^*CDNB*^ and *K*_*m*_^*GSH*^ of SjGST22 were lower than SjGST20, while *K*_*cat*_^*CDNB*^ and *K*_*cat*_^*GSH*^ of SjGST20 were higher than SjGST22. These results showed that SjGST22 had a higher affinity for these two substrates than SjGST20, while SjGST20 had higher catalytic efficiency. For SjGST20, *K*_*cat*_^*CDNB*^ / *K*_*m*_^*CDNB*^ was higher than *K*_*cat*_^*GSH*^ / *K*_*m*_^*GSH*^, which suggested that compared with GSH, CDNB was the optimum substrate. In contrast to SjGST22, GSH was its optimal substrate.

## Discussion

To date, genome-wide *GST* gene identification, bioinformatics analysis, and functional verification have been performed for many plants. However, in *S. japonica*, genome-wide *GST* gene characterization has not been conducted. In this study, a *SjGSTs* gene family of *S. japonica* was identified, and the comprehensive bioinformatics analysis was performed to elucidate features of these genes.

### Sigma GST may be the earliest GST subfamily in *S. japonica*

A total of 32 *SjGST* genes were found, fewer than the number found in *Solanum lycopersicum* (90), *Solanum tuberosum* (90), *Glycine max* (74) and *Camellia sinensis* (88) [11, 21, 35, 36]. In green plants, GSTs are typically divided into eight classes or more [36, 37]. However, SjGSTs only appear in 5 of the 8 clades (Fig. 2), which may indicate a gene loss during the evolution of *S. japonica*.

In plants, phi and tau classes are main GSTs [38]. However, 18 of a total of 32 SjGSTs are sigma class and one is omega class, which two classes more commonly exist in mammals. But this situation is not a special case in brown algae. Roeder et al. (2005) discovered four sigma class GSTs in *L. digitata*, which was the first group of sigma GSTs found in photosynthetic organisms [39]. In the phylogenetic tree of GST sequences from multiple species constructed by Hervé [32], the GSTs of red and brown algae had a closer evolutionary relationship with the sigma GSTs from animals. Referring to the viewpoint of Frova (2006), sigma SjGSTs may appear before eukaryotes differentiation and diverged extensively later in independent lineages [5, 32].

Gene duplication events, which include tandem duplication, segmental duplication, transposition, and whole genome duplication, play a large role in gene family expansion and gene diversity [22, 40]. Groups I and III each contains two tandem repeat gene clusters (Figs. 1 and 3), resulting in significantly more genes in these two groups than in group II. This indicates that tandem duplication is the main expansion mechanism of *SjGSTs*. Replication events mainly occur in the sigma class, indicating that sigma class is the main force driving *SjGST* gene expansion.

Sigma GST is not only the most member in SjGSTs, but also the group with the most tandem duplication, indicating that sigma may be the earliest GST subfamily in *S. japonica*. While members of other classes may later occurrence or gene loss during evolution.

### Multiple SjGSTs help *S. japonica* adapt to the chill growth environment

*SjGSTs* had abundant introns with up to 11 introns found in a gene sequence. There are also multiple introns in *GST* genes of *E. siliculosus* and higher plants [2, 41]. Introns can effectively improve the transcription efficiency of many genes in organisms and can affect transcription by serving as a repository of transcription regulatory elements [42]. Furthermore, alternative splicing is the promoter that generates a variety of structural and functional diversity proteins [43]. Therefore, the abundant introns and alternative splicing sites of *SjGSTs* may promote the functional diversity and expression level of these genes.

CrGST10 (*K*_*m*_^*GSH*^ = 0.32 mM) and CrGST7 (*K*_*m*_^*GSH*^ = 0.31 mM) isolated from *Chlamydomonas reinhardtii* had lower *K*_*m*_^*GSH*^ and higher GSH affinity than SjGST20 and SjGST22 [44]. However, the *K*_*cat*_^*GSH*^ of SjGST20 and SjGST22 were over tenfold greater than CrGST10 (*K*_*cat*_^*GSH*^ = 0.136 s^−1^) and CrGST7 (*K*_*cat*_^*GSH*^ = 1.15 s^−1^), indicating that the catalytic efficiency of SjGST20 and SjGST22 was higher. According to enzyme kinetic parameters, SjGST20 and SjGST22 had different optimal reaction conditions and catalytic substrates, which may mean that when the growth environment of kelp changes, the corresponding *SjGSTs* can respond to stresses more actively and quickly, play an antioxidant activity, or combine with the substrate to play a detoxification role.Same as plant *GSTs* that exhibit constitutive or tissue specific expression patterns, some *SjGSTs* were minimally expressed in all developmental stages and tissues, while some had high expression levels. In addition, other *SjGSTs* were highly expressed in specific stages or tissues. More than half of *SjGST* genes were differentially expressed in different development stages and tissue samples. 24 *SjGSTs* were enriched in glutathione metabolism and arachidonic acid metabolism pathway. These two pathways play a crucial role in resisting cold stress [37]. *S. japonica* is a kind of sub-frigid algae that grows in low temperature seawater. The gene structure, enzymatic characteristics, and metabolic pathway aggregation pattern of *SjGST* are conducive to kelp adaptation to the growth environment and coping with environmental changes.

### *SjGSTs* were important stress resistance genes in *S. japonica*

The stress resistance of plants is closely related to the activity of *GST* to a degree [45]. For instance, many studies have shown that increased *GST* gene expression in chickpea, barley, and wheat is positively correlated with improved stress tolerance [46–48]. Heterologous expression of the *Juglans regia GST* gene in tobacco can effectively reduce ROS produced in response to low temperatures and improve the cold tolerance of transgenic tobacco [49]. Two main kinds of *cis*-acting elements are found in SjGST promoter regions, including light, temperature, drought, anaerobic and stress respond elements and multiple plant hormones respond elements, indicate that *SjGSTs* are induced by these environmental and hormonal signals. In this research, when *S. japonica* sporophytes were stressed by high temperature, low salt, and drought, the expression of *SjGST4* and *SjGST20* increase significantly. Heterologous expression of *SjGSTs* in *S. cerevisiae* increased the growth rate of recombinant yeast under NaCl and H_2_O_2_ stresses. Previous studies have also reported the response of GST to these signals. For example, *CmGSTs* actively responded to cold stress in *Cucumis melo* [50]. *PtGSTF1* improved transgenetic *Populus trichocarpa* salt tolerance [51]. These results illustrate *SjGSTs* are important stress resistance genes in *S. japonica*, regulating the responds of algae to stresses.

## Conclusion

In this study, we performed a genome-wide analysis of the *SjGST* gene family in *S. japonica*; a total of 32 genes categorized into three groups were identified. Further, the characteristics of these genes were analyzed, including sequence features, scaffold location, phylogenetic relationship, gene duplication, *cis-*acting elements in promoter regions, developmental stage- and tissue-specific expression patterns, and dynamic transcription profiles in response to low salinity, drought, and high temperature. Additionally, three *SjGSTs* were isolated and recombined into *S. cerevisiae*, which promoted the growth rate of recombinant yeasts under stress conditions. Finally, the optimum temperature and pH, *K*_*m*_, *V*_*max*_, *K*_*cat*_ and *K*_*cat*_ /*K*_*m*_ for CDNB and GSH of SjGST20 and SjGST22 were determined. Our research shows that *SjGSTs* are important stress resistant genes in *S. japonica*, which can help kelp adapt to cold growth environments and have a positive response to salinity, drought, hydrogen peroxide, and temperature stresses. This research lays the foundation for future functional verification of the *SjGST* genes in abiotic stress resistance.

## Methods and materials

### Algal sample collection and culture conditions

*S. japonica* was collected from cultivated rafts in Rongcheng, Shandong, China. For low salinity and drought treatments, juvenile sporophytes collected on 8 December 2019 were used. For high temperature treatments, juvenile sporophytes collected on 5 December 2020 were used. The sporophytes receiving stress treatments were pre-cultured in the dark overnight at 10 °C. Due to the inability to completely restore the natural growth environment in the laboratory, long-term laboratory culture has a negative impact on the physiological status of *S. japonica*. Therefore, the s sporophytes were subjected to short-term stress treatment.

To examine salinity stress, the sporophytes were cultured at salinities of 32‰, 24‰, 16‰, and 8‰ for 1.5 h. For drought stress, sporophytes were exposed to air for 0 h, 0.5 h, 1 h, 1.5 h, 2 h, and 2.5 h. For high temperature stress, sporophytes were cultured in seawater at 25 °C for 0 h, 0.5 h, 1 h,1.5 h, 2 h, and 2.5 h. Three individual replicates were used for each test, and each tissue sample was frozen in liquid nitrogen and stored at -80 °C for subsequent RNA isolation.

### Genome and transcriptome data analysis of *S. japonica*

Our previous genome data can be found in GenBank (National Center for Biotechnology Information, NCBI) with the accession number MEHQ00000000.1 (https://www.ncbi.nlm.nih.gov/nuccore/MEHQ00000000.1/). The treated sporophytes used for RNA-seq analysis were collected in January, March, April, May, and June. The sporophytes collected in April were used for RNA-seq analysis of the kelp basal blade, 1/3 blade, 2/3 blade, and distal blade. All resulting transcriptome data were registered in the NCBI Sequence Read Archive (SRA) with an accession number PRJNA512328 (https://www.ncbi.nlm.nih.gov/bioproject/PRJNA512328). The sequencing, assembly, and annotation methods of the *S. japonica* transcriptome were described in detail by Shao et al. [52].

### Identification of *SjGST* genes and its family features

We searched in the transcriptome sequence annotation files using the keyword “glutathione S-transferase”. If the gene annotation result contained “glutathione S-transferase”, this gene was eligible for selection as a candidate gene for further identification. All candidate genes were submitted to NCBI Covserved Domain (https://www.ncbi.nlm.nih.gov/Structure/cdd/wrpsb.cg), MOTIF (https://www.genome.jp/tools/motif/) and Pfam (http://pfam.xfam.org/search) to confirm the presence of the conserved domain with cut-off scores of E-value < 0.05; only genes that encode glutathione S-transferase domains were retained [53, 54]. We followed the methods of Lu et al. (2020) for sequence analysis, scaffold localization, and gene duplication analysis [55].

The 32 identified *S. japonica* GST proteins were aligned by multiple alignments using fast Fourier transform (MAFFT) with the default parameters, and secondary structures were visualized using Easy Sequencing in PostScript (ESPript) 3.0 [56, 57]. To analyze the evolutionary relationships among the 32 SjGSTs in *S. japonica*, a maximum likelihood (ML) phylogenetic tree was constructed based on the full-length amino acid sequences with MEGA 7.0.26 using the WAG + G model with 1000 bootstrap replications, Gamma 2, partial deletion, and 50% site coverage as the cutoff value [58].

### Sequence alignment and phylogenetic analysis

Phylogenetic analysis was performed on the amino acid sequences of GSTs derived from *E. siliculosus* (12), *C. okamuranus* (19), *N. decipiens* (9),* L. digitata* (4), *P. tricornutum* (10),* T. weissflogii* (7), *A. thaliana* (13) and *S. japonica* (32). Details and amino acid sequences on the 106 sequences are provided in Additional file 4: Table S4. The maximum likelihood (ML) phylogenetic tree was constructed from the full-length amino acid sequences of the 106 GST proteins using MEGA 7.0.26 with 1000 bootstrap replications, the WAG + G model, Gamma 7, partial deletion, and 50% site coverage as the cutoff value [58]. For reciprocal BLAST, SjGSTs were used as queries against SjGSTs, EsGSTs, LdGSTs, CoGSTs, NdGSTs, PtGSTs, TwGSTs and AtGSTs, respectively. The program ran in TBtools and local BLAST, and the parameter settings were as follows: Number of retained hits, 1; E-value, 1E-5; minimum weighted coverage, 0.33.

### Identification of alternative splicing events

Tophat 2.1.1 was used to analyze alternative splicing events in the SjGSTs from RNA-Seq data. Alternative splicing sites supported by less than five reads were filtered out, and the remaining were mapped to known alternative splicing sites, allowing for 1 bp error. The known alternative splicing sites were identified and the unmapped new alternative splicing sites were classified again. The junction structure and classification introduction are listed in the Additional file 5: Table S5 and Additional file 12: Figure S3.

### Promoter region cis-regulatory element analysis of *SjGSTs*

Upstream genomic sequences within 2 kb of the start codons of all *SjGSTs* were extracted from the genome of *S. japonica*. *Cis-*acting elements in the putative promoter regions were identified via the plant *cis*-acting regulatory elements (PlantCARE) database (http://bioinformatics.psb.ugent.be/webtools/plantcare/html/) [59].

### cDNA synthesis and qRT-PCR analysis of SjGSTs

Total *S. japonica* RNA extraction and first-strand cDNA synthesis followed Lu (2020) [55]. cDNA was stored at − 20 °C for subsequent analysis. Gene-specific primers used for qRT-PCR are listed in Table 5. qRT-PCR was performed on a Takara Thermal Cycle Dice™ Real-Time System (Takara, Japan). Conditions used for qRT-PCR were as follows: 94 °C for 2 min 30 s; 40 cycles of 94 °C for 15 s, 55 °C for 30 s, and 72 °C for 25 s; and one cycle of 95 °C for 15 s, 60 °C for 60 s, and 72 °C for 15 s. Three biological replicates were performed. Reaction mixtures, internal control, and relative transcriptional levels calculation method referred the protocols of Lu et al. (2020) [55]. SPSS 26.0 was used for statistical analysis.

**Table 5:** Primers used for qRT-PCR

Gene	Forward primer (5′-3′)	Reverse primer (5′-3′)
*β-actin*	GACGGGTAAGGAAGAACGG	GGGACAACCAAAACAAGGGCAGGAT
*SjGST4*	CTCGTACTTCCCGTTCCTCG	CCAGCCCTCACGAAGTAGTC
*SjGST20*	ATAGAGGACATCGCCAGCAAG	CTTCACCTTGGGGTGTTCCAT
*SjGST22*	GAATTTGGCGCTCTCAAGCC	GTCGTCGGAAGGGTACAGTC

### PCR amplification and sequencing of the *GST* genes

Comparing the results of gel electrophoresis and cloning sequencing with other SjGSTs, the PCR amplification product of *SjGST4*, *SjGST20* and *SjGST22* had the best quality. Therefore, we would conduct follow-up researches based on these three genes. Primers used to amplify the full-length cDNA sequences of these three genes are listed in Table 6. Reaction mixtures of 20 μL contained 10 μL 2 × Phanta Max Master Mix (Vazyme, China), 2 μL *S. japonica* cDNA as the template, 1 μL of each of the forward and reverse primers (10 μM), and 6 μL ddH_2_O. Conditions used for PCR were as follows: 98 °C for 3 min; 40 cycles of 98 °C for 15 s, 60 °C for 20 s, and 72 °C for 30 s; and one cycle of 72 °C for 10 min.

**Table 6:** Primers used for gene cloning

Gene	Forward primer (5′-3′)	Reverse primer (5′-3′)
*SjGST4*	CGCGGATCCATGTCTGCAACCACTCTGAG	CCGGAATTCCTTCTTGGCGACCCCAGCCC
*SjGST20*	CGCGGATCCATGGTTCCCGTATTTAACTA	CCGGAATTCCGCCTTGGAGGCGTAGTACG
*SjGST22*	CGCGGATCCATGGCTCCCAAGTTGGTCCT	CCGGAATTCCTTGGCGTGCTTGGCGATGA

The PCR products were inserted into a TOPO cloning vector using the 5 min TA/Blunt-Zero Cloning Kit (Vazyme, China) according to the protocol of the manufacturer. Recombinant plasmids were transformed into TSINGKE DH5α Chemically Competent Cells (TSINGKE, China) and then Sanger sequenced by Sangon Biotech (Sangon, China).

### Heterologous expression of recombinant protein in *Escherichia coli*

For prokaryotic expression in *Escherichia coli*, we used pET-32a for overexpression of *SjGST20* and *SjGST22.* Recombinant vectors containing the target genes were transformed into BL21(DE3) pLysS competent cells (TSINGKE, China).

Recombinant strains appearing as white single colonies on LB (Luria broth) agar plates (Amp^+^) were selected and transferred into 1 mL LB (Amp^+^ and Chl^+^). The recombinant strains were inoculated into 20 mL LB (Amp^+^ and Chl^+^) and incubated overnight at 37 °C. The 20 mL bacterial suspension was added to 1 L LB (Amp^+^ and Chl^+^) at a 1:50 ratio for secondary incubations. When the OD_600_ reached 0.6–0.8, the culture medium was placed in ice water for 30 min and was induced by adding 0.25 mM isopropyl-β-D-thiogalactopyranoside (IPTG) at 18 °C with shaking at 100 rpm for 20 h.

### Purification of recombinant proteins

The recombinant proteins expressed in *E. coli* were purified for the detection of enzyme activities. The bacterial precipitate from centrifuging 1 L LB was resuspended with binding buffer. The resuspended bacterial precipitate was disrupted by ultrasonication (cycles of ultrasonic crushing for 3 s, pausing for 6 s; the total time for the procedure was 30 min) and then centrifuged to collect the supernatant. The recombinant protein with His-tag was specifically adsorbed onto a His-Ni^2+^ column (Cytiva, United States) and was eluted by elution buffer. The binding buffer contained 0.1 M potassium phosphate, 30 mM imidazole, 5% (v/v) glycerol, and 150 mM sodium chloride, pH 7.0. For the elution buffer, the concentration of imidazole was increased to 500 mM, the concentrations of other components were the same as that of binding buffer.

### Induced expression and stress culture of recombinant yeast

For *S. cerevisiae* expression, we used pYES2-CT for overexpression of *SjGST4*, *SjGST20*, and *SjGST22*. Recombinant vectors containing the target genes were transformed into INVSc1 competent cells (Coolaber, China). Synthetic dextrose (SD)-Ura nutrient-deficient plates were used for screening the recombinant yeast.The tolerance of recombinant yeast was detected with reference to the method of Patankar et al. (2019) and adjusted according to the actual situation [60]. Recombinant strains appearing as white single colonies on SD-Ura nutrient-deficient plates were selected and transferred into 1 mL yeast extract-peptone-dextrose (YPD) medium (with glucose as the carbon source). Recombinant yeasts were cultured at 29 °C overnight. Then, the medium was changed to 5 mL YPD (with galactose as the carbon source) to induce the expression of the *SjGSTs*. Recombinant yeasts were cultured at 29 °C for two days.

Induced recombinant yeasts solutions were diluted to OD_600_ of approximately1.0 to produce the primary bacterial suspension. Then, the primary bacterial suspension was diluted 10, 100, and 1000 times. To the plates used for stress treatments, 5 μL of each bacterial suspension was successively added, and plates were cultured upside down at 29 °C for four days.

The basic YPD plate contained 2 g tryptone, 1 g yeast extract, 0.004 g adenine sulfate, 2 g galactose, 1.5 g agar and 100 mL ddH_2_O. For salinity stress, sodium chloride was added to basic YPD to final concentrations of 0.9 M, 1.1 M, 1.3 M, and 1.5 M. For H_2_O_2_ stress, H_2_O_2_ was added to basic YPD to final concentrations of 1 mM, 3 mM, 5 mM, 7 mM, and 8 mM. The growth of recombinant yeast on plates without sodium chloride and H_2_O_2_ was used as the control.

For temperature stress, 5 μL of bacterial suspension was added to basic YPD plates, and plates were cultured upside down at 25 °C, 35 °C, 37 °C and 40 °C for three days.

### In vitro GST assays

The enzymatic activity of GST was detected with reference to the method of Mejia-Sanchez et al. (2018) and adjusted according to the actual situation [61]. In brief, to test the optimal reaction temperature of recombinant enzymes, a total assay volume of 300 μL was used consisting of 10 μL 1-chloro-2, 4-dinitrobenzene (CDNB, 60 mM), 10 μL glutathione (GSH, 60 mM), 10 μL recombinant enzyme, and 270 μL potassium phosphate buffer (0.1 M, pH = 7.0). The assay solutions were mixed and incubated for 5 min at 5 °C, 10 °C, 15 °C, 20 °C, 25 °C, 30℃, and 35℃. Reactions were terminated by adding 10 μL HCl (6 M) to the mixtures. The formation of S-(2, 4-dinitrophenyl)-glutathione (GS-DNB) was detected at an absorbance of 340 nm.

For the optimal pH of recombinant enzymes, a total assay volume of 900 μL was used consisting of 30 μL CDNB (60 mM), 30 μL GSH (60 mM), 10 μL recombinant enzyme, and 830 μL potassium phosphate buffer (0.1 M, pH = 5.0, 5.5, 6.0, 6.5, 7.0, 7.5, 8.0, 8.5, and 9.0). The assay solutions were mixed and incubated for 5 min at 20 °C. To terminate the reactions, 20 μL HCl (6 M) was added to the mixtures. The formation of GS-DNB was detected at an absorbance of 340 nm.

*K*_*m*_ values for GSH were determined using 30 μL CDNB (60 mM) and 30 μL GSH at concentrations of 60 mM, 30 mM, 20 mM, 15 mM, 10 mM, 6 mM, and 3 mM. *K*_*m*_ values for CDNB were determined using 30 μL GSH (60 mM) and 30 μL CDNB at concentrations of 60 mM, 30 mM, 20 mM, 15 mM, 10 mM, 6 mM, and 3 mM. The assays also consisted of 15 μL recombinant enzyme and 825 μL potassium phosphate buffer (0.1 M). The *K*_*m*_ values of recombinant enzymes were determined at 20 °C in buffer at pH 7.0 for SjGST20 and at 20 °C in buffer at pH 8.0 for SjGST22. To terminate the reactions, 20 μL HCl (6 M) was added to the mixtures.

### Supplementary Information

**Additional file 1: Supplementary Table S1.** Subcellular localization prediction of the SjGSTs.

**Additional file 2: Supplementary Table S2.** Reciprocal BLAST using the candidate SjGST proteins as queries against *Saccharina japonica* SjGST proteins. **Supplementary Table S2.** Reciprocal BLAST using the candidate SjGST proteins as queries against *Laminaria digitata* LdGST proteins. **Supplementary Table S2.** Reciprocal BLAST using the candidate SjGST proteins as queries against *Ectocarpus siliculosus* EsGST proteins. **Supplementary Table S2.** Reciprocal BLAST using the candidate SjGST proteins as queries against *Cladosiphon okamuranus* CoGST proteins. **Supplementary Table S2.** Reciprocal BLAST using the candidate SjGST proteins as queries against *Nemacystus decipiens* NdGST proteins. **Supplementary Table S2.** Reciprocal BLAST using the candidate SjGST proteins as queries against *Phaeodactylum tricornutum* PtGST proteins. **Supplementary Table S2.** Reciprocal BLAST using the candidate SjGST proteins as queries against *Thalassiosira weissflogii* TwGST proteins. **Supplementary Table S2.** Reciprocal BLAST using the candidate SjGST proteins as queries against *Arabidopsis thaliana* AtGST proteins.

**Additional file 3: Supplementary Table S3.** The putative conserved motifs of SjGSTs.

**Additional file 4: Supplementary Table S4.** GST amino acids sequences from all the selected species for phylogenetic analysis. **Supplementary Table S4.** NCBI accession number of GSTs. **Supplementary Table S4.** Sub-family classes of GSTs.

**Additional file 5: Supplementary Table S5.** The types of alternative splicing sites in *SjGST*.

**Additional file 6: Supplementary Table S6.** The secondary structure features of SjGSTs.

**Additional file 7: Supplementary Table S7.** Transcriptional profiles enriched pathway in *S. japonica* different development stages.

**Additional file 8: Supplementary Table S8.** Transcriptional profiles enriched pathway in *S. japonica* different tissues.

**Additional file 9: Supplementary Table S9.** The KEGG pathway of SjGSTs.

**Additional file 10: Fig. S1.** SDS-PAGE results of recombinant SjGST20 and SjGST22. 1: protein ladder, 2: expression of SjGST20 induced with 0.25 mM IPTG, 3: purified SjGST20 fusion protein, 4: protein ladder (same as 1), 5: expression of SjGST22 induced with 0.25 mM IPTG, 6: purified SjGST22 fusion protein.

**Additional file 11: Fig. S2.** Western blot results of recombinant SjGST20 and SjGST22. 1: protein ladder, 2: expression of SjGST20 induced with 0.25 mM IPTG, 3: purified SjGST20 fusion protein, 4: protein ladder (same as 1), 5: expression of SjGST22 induced with 0.25 mM IPTG, 6: purified SjGST22 fusion protein.

**Additional file 12: Fig S3.** Structure of junction.

## Data Availability

https://www.ncbi.nlm.nih.gov/genbank/ https://marinegenomics.oist.jp/gallery/ https://www.ncbi.nlm.nih.gov/nuccore/MEHQ00000000.1/ https://www.ncbi.nlm.nih.gov/bioproject/PRJNA512328

## Abbreviations

AS: Alternative splicing
CDNB: 1-Chloro-2,4-dinitrobenzene
ES: Exon skipping
GS-DNB: S-(2, 4-dinitrophenyl)-glutathione
GSH: Glutathione
GST: Glutathione S-transferase
IR: Intron retention
KEGG: Kyoto Encyclopedia of Genes and Genomes
MAFFT: Multiple alignments using fast Fourier transform
MeJA: Methyl jasmonate
ML: Maximum likelihood
MW: Molecular weight
NCBI: National Center for Biotechnology Information
ORF: Open reading frame
pI: Isoelectric point
ROS: Reactive oxygen species
TSS: Transcription start sites
TTS: Transcription terminal sites
UTRs: Untranslated regions
