# Supplementary material for: Isolation and characterization of glutathione S-transferase genes and their transcripts in Saccharina japonica (Laminariales, Phaeophyceae) during development and under abiotic stress
Source: BMC Plant Biol. 2023 Sep 18;23:436. doi: 10.1186/s12870-023-04430-5 (PMC10506224; doi:10.1186/s12870-023-04430-5)
Supplement: Supplementary file 10 — Additional file 10: Fig. S1. SDS-PAGE results of recombinant SjGST20 and SjGST22. 1: protein ladder, 2: expression of SjGST20 induced with 0.25 mM IPTG, 3: purified SjGST20 fusion protein, 4: protein ladder (same as 1), 5: expression of SjGST22 induced with 0.25 mM IPTG, 6: purified SjGST22 fusion protein. [file 12870_2023_4430_MOESM10_ESM.pdf]

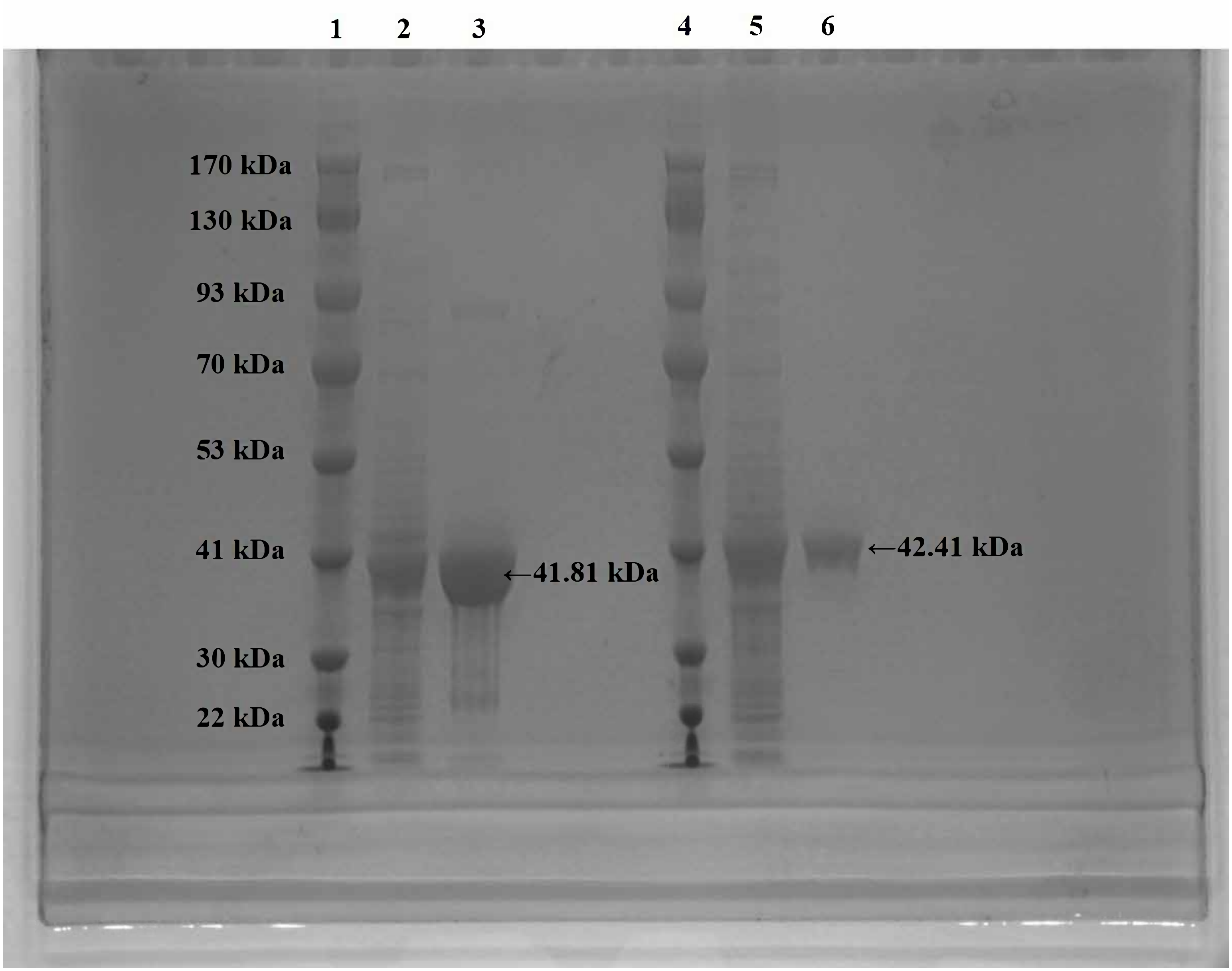

Fig. S1 SDS-PAGE results of recombinant SjGST20 and SjGST22. 1: protein ladder, 2: expression of SjGST20 induced with 0.25 mM IPTG, 3: purified SjGST20 fusion protein, 4: protein ladder (same as 1), 5: expression of SjGST22 induced with 0.25 mM IPTG, 6: purified SjGST22 fusion protein
