# Supplementary figures and images for: Isolation and characterization of glutathione S-transferase genes and their transcripts in Saccharina japonica (Laminariales, Phaeophyceae) during development and under abiotic stress
Source: BMC Plant Biol. 2023 Sep 18;23:436. doi: 10.1186/s12870-023-04430-5 (PMC10506224; doi:10.1186/s12870-023-04430-5)

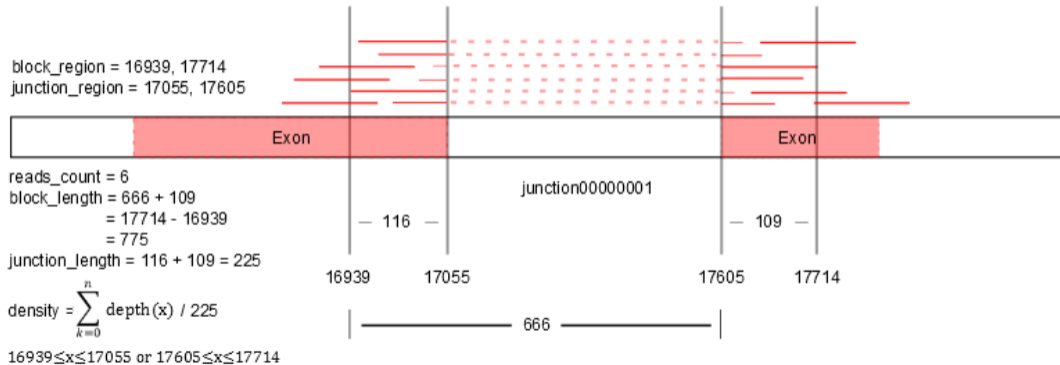

**Fig S3 Structure of junction**

Supplement: Supplementary file 12 — Additional file 12: Fig S3. Structure of junction. [file 12870_2023_4430_MOESM12_ESM.pdf]
